# Biological Activities of 2,3,5,4′-Tetrahydroxystilbene-2-O-*β*-D-Glucoside in Antiaging and Antiaging-Related Disease Treatments

**DOI:** 10.1155/2016/4973239

**Published:** 2016-06-20

**Authors:** Shuang Ling, Jin-Wen Xu

**Affiliations:** Murad Research Institute for Modernized Chinese Medicine, Shanghai University of Traditional Chinese Medicine, Shanghai 201203, China

## Abstract

2,3,5,4′-Tetrahydroxystilbene-2-O-*β*-D-glucoside (THSG) is active component of the Chinese medicinal plant* Polygonum multiflorum* Thunb. (THSG). Pharmacological studies have demonstrated that THSG exhibits numerous biological functions in treating atherosclerosis, lipid metabolism, vascular and cardiac remodeling, vascular fibrosis, cardiac-cerebral ischemia, learning and memory disorders, neuroinflammation, Alzheimer and Parkinson diseases, diabetic complications, hair growth problems, and numerous other conditions. This review focuses on the biological effects of THSG in antiaging and antiaging-related disease treatments and discusses its molecular mechanisms.

## 1. Introduction

Aging is inevitable; it is a progressive, irreversible process that every human will experience in his life. The aging population of the international community brings increasing medical expenses and health care costs. Therefore, prevention and early treatment of aging-related diseases can be effective means of relieving society's burden and living a better life for individuals. There are many theory researches of aging mechanisms. The most famous one is the oxidative stress theory. Free radicals and peroxides attack all components of cells, including proteins, lipids, RNA, and DNA. Oxidative damage occurs in various aging-associated disease pathologies, especially the cardiovascular diseases and neurological diseases. Theoretically, antioxidant supplementation should be able to reduce the risk of aging-related diseases. The Mediterranean diet with red wine, fruits, vegetables, and other plant foods has been shown to have cardiovascular protection against oxidative damage. At present, the extraction of biological antioxidants from plants is becoming one of the hot topics in the field of medical chemistry.

**Figure 1:**
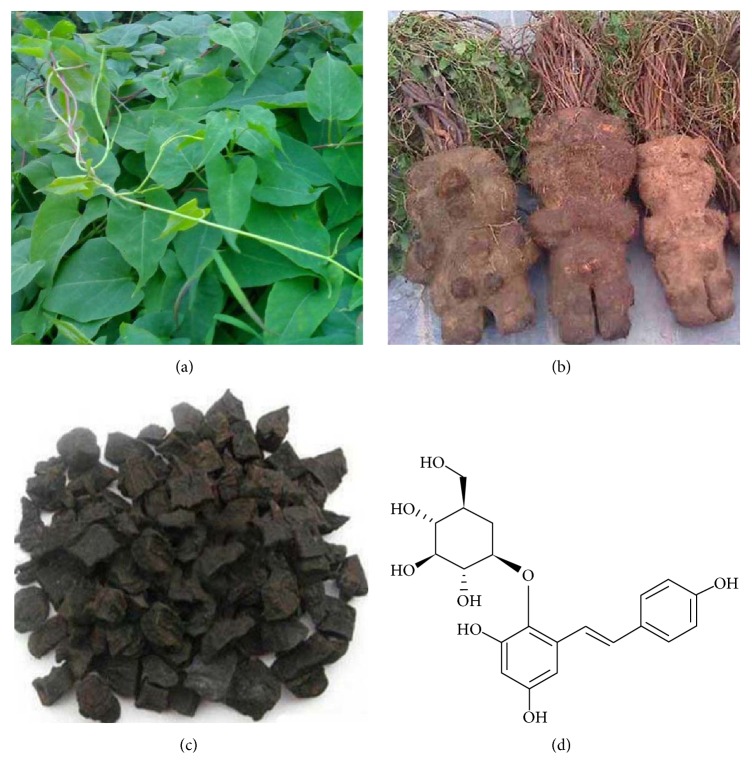
The images of medicinal material* Polygonum multiflorum* and molecular structure of THSG. (a) Seedling herbs, (b) harvested herbs, (c) processed herbs, radix* Polygoni Multiflori* preparata, and (d) chemical structure of THSG.

*Polygonum multiflorum* Thunb. (何*首乌*,* he-shou-wu*) (Figures 1(a) and 1(b)) is a traditional Chinese medicinal plant. As early as 973 A.D., it was incorporated into* Kaibao Bencao*, an encyclopedia of medical plants edited under an imperial edict of Song Taizu, the first emperor of the Song Dynasty. The plant is processed to product radix* Polygoni Multiflori* preparata (Figure 1(c)), traditionally taken to increase vitality, improve the health of blood and blood vessels, blacken hair, strengthen bones, nourish the liver and kidney, and prolong life. Currently,* Polygonum multiflorum* Thunb. is listed in the* Chinese Pharmacopoeia*, and radix* Polygoni Multiflori* preparata is widely used for clinically treating of arteriosclerosis, hyperlipidemia, hypercholesterolemia, and diabetes. It is also used in many Chinese medicinal supplements to improve general health.

2,3,5,4′-Tetrahydroxystilbene-2-O-*β*-D-glucoside (THSG) (Figure 1(d)) is the main component of* Polygonum multiflorum* Thunb., which is used as a standard compound for appraising* Polygonum multiflorum* Thunb. in the* Chinese Pharmacopoeia* [1]. THSG belongs to polyhydroxystilbene group. The structure of THSG is similar to that of resveratrol (3,4′,5-Trihydroxy-trans-stilbene), which is quite well known for its numerous biological activities especially in cardiovascular protection. As a resveratrol analog with glucoside, THSG has been proved to possess strong antioxidant and free radical scavenging activities even much stronger than resveratrol in superoxide anion radical scavenging, hydroxyl radical scavenging, and DPPH radical scavenging [2]. It is because THSG has a 2-O-Glu group in chemical structure, in which C_5_-OH and C_4_′-OH are more active to H-abstraction [3]. Furthermore, 2-O-Glu group can stabilize the phenoxyl free radicals and they are easy to be hydrolyzed in extreme pH environments (in the gastrointestinal environment).

Contemporary pharmacological studies have demonstrated that THSG exhibits numerous biological functions in antiaging and antiaging-related disease treatments. In this review, we focus on THSG, discussing its biological effects and molecular mechanisms.

## 2. Delaying the Senescence Effect

A few years ago, we found that THSG can delay vascular senescence and markedly enhance blood flow in spontaneously hypertensive rats (SHRs), but it does not affect blood pressure or body weight [4]. The data revealed that senescence-associated *β*-galactosidase (SA-*β*-gal) staining, *γ*H2AX phosphorylation, and p53 acetylation are suppressed by THSG in the aortic arches of SHRs. THSG promotes deacetylation of p53, a transcription factor associated with aging. THSG also induces endothelial nitric oxide synthase (eNOS) expression in the aortas and urinary mononitrogen oxide (NO_*x*_) production.* In vitro*, THSG activates SIRT1 activity, stimulates eNOS promoter reporter gene activity, and ameliorates H_2_O_2_-induced human umbilical vein endothelial cell (HUVEC) senescence [4]. Our unpublished data show that* in vivo* THSG is more effective in delaying vascular senescence than resveratrol.

A recent study revealed that THSG prolongs the lifespan of senescence-accelerated prone mouse (SAMP8) by 17% and notably improves their memory. THSG also increase neural klotho protein level and reduce levels of the neural insulin, the insulin receptors, insulin-like growth factor-1 (IGF-1), and IGF-1 receptor in the brain of SAMP8 [5]. In a subsequent report, this research group again demonstrated that THSG improves memory, reduces levels of reactive oxygen species (ROS), nitric oxide (NO), and IGF-1, and increases protein levels of superoxide dismutase (SOD) and klotho in serum. Furthermore, THSG upregulates klotho protein expression in cerebrum, heart, kidney, testis, and epididymis tissues of D-galactose induced aging mice [6].

A German study reported that THSG exerted a DAF-16-independent antiaging effect in a* Caenorhabditis elegans* model [7]. THSG prolongs the mean, median, and maximum adult lifespans of* C. elegans* by 23.5%, 29.4%, and 7.2%, respectively, and increases the resistance of* C. elegans* to lethal thermal stress, comparable to the effects of resveratrol. THSG also exerts a higher antioxidative capacity in nematode compared with resveratrol and reduces the levels of the aging pigment lipofuscin.

## 3. Cardiovascular Protection

### 3.1. Atherosclerosis and Lipid Metabolism

An experimental investigation using New Zealand rabbits demonstrated that THSG reduces atherosclerotic plaque accumulation caused by a high cholesterol diet, and lower plasma cholesterol, low-density lipoprotein (LDL) cholesterol, very-low-density lipoprotein (VLDL) cholesterol, and triglyceride levels [8]. Moreover, THSG decreases secretion protein levels of the intercellular adhesion molecule- (ICAM-) 1 and the vascular endothelial growth factor (VEGF) in the U937 foam cell cultured medium [8]. Subsequent studies have reported that in rat aortic walls in high-cholesterol-fed rats THSG improves the serum lipid profile and suppresses serum C-reactive protein (CRP), IL-6 and TNF-*α* levels, and matrix metalloproteinase- (MMP-) 2, MMP-9 mRNA, and protein expressions [9]. THSG also restores the mRNA and protein expression of eNOS in the rat aorta and improves acetylcholine-induced endothelium-dependent relaxation [10]. THSG exhibited antioxidant properties and protected against apoptosis in a lysophosphatidylcholine- (LPC-) induced endothelial cell injury model [11]. THSG suppresses intracellular ROS and malondialdehyde (MDA) and restores SOD and glutathione peroxidase (GSH-Px) levels. THSG apparently reversed the loss of mitochondrial membrane potential, the activation of caspase-3 and poly(ADP-ribose) polymerase 1 (PARP-1), the decrease of Bcl-2, the upregulation of Bax, and the release of cytochrome C in LPC-stimulated HUVECs [11].

Ten years ago, a Japanese group found that THSG does not affect the food intake, growth, or blood pressure of SHRs, consistent with our data [4, 12], but significantly reduces free fatty acid content in serum. THSG significantly reduces cholesterol and neutral lipid content in the VLDL fraction and neutral lipid content in the high-density lipoprotein (HDL) fraction in the blood, as well as neutral lipid content in the liver [12]. Another study reported that THSG administration to rats for 1 week can effectively control serum levels of total cholesterol and LDL cholesterol. The expression of LDL receptors in the liver was significantly upregulated in a high-fat-fed rat model [13]. Furthermore,* in vitro* experiments revealed a downregulation effect of THSG on 3-hydroxy-3-methylglutaryl-coenzyme A (HMG-CoA) reductase and an upregulation effect on cholesterol 7 alpha-hydroxylase (CYP7A) in human steatosis L02 cells. THSG enhanced downregulation activities in TC, LDL cholesterol, and VLDL contents and increased activity in HDL cholesterol [14].

### 3.2. Vascular Remodeling and Fibrosis

*In vitro*, THSG prevents the proliferation of vascular smooth muscle cells (VSMCs) and blocks the G1/S phase progression of the cell cycle in platelet-derived growth factor-BB- (PDGF-BB-) or angiotensin II-induced VSMCs [15, 16]. THSG inhibits the phosphorylation of Rb and extracellular signal-regulated kinase 1/2 (ERK1/2); it also inhibits the expressions of cyclin D1, cyclin-dependent kinase-4 (CDK4), CDK2, cyclin E, the proliferating cell nuclear antigen (PCNA) in PDGF-BB-induced VSMCs [15], phosphorylated ERK1/2, MEK1/2, Src, c-fos, c-jun, and c-myc mRNA in angiotensin II-induced VSMCs [16].* In vivo*, THSG inhibits neointimal hyperplasia in a rat carotid arterial balloon injury model [17], and the ratio of intima-to-media was significantly reduced, and the expressions of PCNA, *α*-smooth muscle actin (*α*-SMA), and PDGF-BB were suppressed. Moreover, signaling pathways associated with smooth muscle cell proliferation, migration, and inflammation were inhibited, in addition to the activation of AKT, ERK1/2, and nuclear factor *κ*B (NF-*κ*B) and the expressions of c-myc, c-fos, c-jun, MMP-2, MMP-9, and collagens I and III [17]. Our recent study reported that orally administering THSG for 14 weeks significantly inhibited vascular remodeling and fibrosis in SHRs with increasing blood flow and with constant blood pressure [18]. THSG reduces intima-media thickness in the aortic arch of SHRs, increases the vascular diastolic rate in response to acetylcholine, and reduces remodeling and fibrosis-related mRNA expression, such as that of genes* ACTA2*,* CCL3*,* COL1A2*,* COL3A1*,* TIMP1 WISP2*,* IGFBP1*,* ECE1*,* KLF5*,* MYL1 BMP4*,* FN1*, and the plasminogen activator inhibitor-1 (*PAI-1*). THSG inhibits the acetylation of Smad3 and prevents Smad3 binding to the PAI-1 proximal promoter in SHR aortas [18].

### 3.3. Heart

THSG improves cardiac ischemia-reperfusion, cardiac remodeling, and cardiac stem cells. The infarct size, ST segment recovery, and incidence of arrhythmia in the THSG postconditioning group are all significantly improved compared with the control group [19]. THSG has also been shown to promote mitochondrial biogenesis and induce the expression of erythropoietin (EPO) in nonhematopoietic cells, including primary cardiomyocytes, and enhance EPO–EPO receptor autocrine activity. THSG robustly increases the endurance performance activity of healthy and doxorubicin-induced cardiomyopathic mice in ischemic disorders, stimulates myocardial mitochondrial biogenesis, and improves cardiac function [20].

In cardiac remodeling, THSG can attenuate pressure overload-induced cardiac pathological changes. Such pathological changes include increases in heart weight/body weight and left ventricular weight/body weight ratios, increased myocyte cross-sectional areas and left ventricular posterior wall, hypertrophic ventricular septum, and accumulation of myocardial interstitial perivascular collagen, as well as elevated cardiac hydroxyproline content [21]. Furthermore, THSG significantly reduces myocardium angiotensin II, enhances the activities of SOD and GSH-Px in serum and myocardial tissue, and inhibits the protein expression of transforming growth factor beta 1 (TGF-*β*1) and the phosphorylation of ERK1/2 and p38 MAP kinase in myocardial tissue [22]. However, THSG treatment increases the percentage of the S-phase in sorted c-kit(+) rat cardiac stem cells and promotes expressions of PCNA, VEGF, the T-box transcription factor, hyperpolarization-activated cyclic nucleotide-gated 2 (HCN2), HCN4, the *α* myosin heavy chain, *β* myosin heavy chain mRNA, stem cell antigen 1, cardiac troponin-I, GATA-4, Nkx2.5, and connexin 43 protein [22].

### 3.4. Platelets

*In vitro*, THSG treatment inhibits adenosine diphosphate- (ADP-) or thrombin-induced platelet aggregation dose-dependently. THSG does not affect intracellular calcium ion dynamics at rest; however, in the ADP or thrombin stimulation, THSG reduces dose-dependently the rise in intracellular calcium flow [23]. Another study demonstrated that THSG prevents dose-dependently collagen-induced platelet aggregation and ATP secretion [24]. THSG also inhibits platelet P-selectin expression, glycoprotein IIb-IIIa binding, and platelet spreading on immobilized fibrinogen, as well as Fc receptor Fc*γ*RIIa, Akt (Ser473), and GSK3*β* (Ser9) phosphorylations [24].

## 4. Neuroprotective Effects

### 4.1. Learning and Memory

In *β*-amyloid peptide-induced dementia mice, ischemia-reperfusion gerbils, and D-galactose induced dementia mouse models, oral administration of THSG for dementia prevention or treatment improves learning and memory function in Morris water maze tests. THSG significantly decreases MDA level and monoamine oxidase B activity in the cerebral cortex, reduces the affinity of NMDA receptors with ^3^H-MK801, and increases expression of nerve growth factor (NGF) and neurotrophic factor-3 in the hippocampal CA1 region [25–27]. Moreover, THSG promotes the differentiation of PC12 cells, increases the intracellular calcium level in hippocampal neurons, and facilitates high-frequency stimulation-induced hippocampal long-term potentiation (LTP) in a bell-shaped manner. The facilitation of LTP induction by THSG required calcium/calmodulin-dependent protein kinase II and ERK activation [28].* In vivo*, THSG treatment also restores memory impairment, as assessed using the passive avoidance test, in models for sleep-deprived mice, amyloid-*β*-injected aging mice, and kainic acid-injected brain-damage mice. Concurrently, THSG induces expressions of erythropoietin, PPAR-*γ* coactivator 1*α* (PGC-1*α*), and hemoglobin in astrocytes and PC12 neuronal-like cells and in the hippocampus of mice [29].

### 4.2. Neuroinflammation

Neuroinflammation is closely implicated in the pathogenesis of neurological diseases. Thus, the inhibition of microglial inflammation may have potential therapeutic significance for neurological diseases. Researchers have used a microglia BV2 cell line as a model to investigate the antineuroinflammatory effects of THSG, finding that THSG reduced the LPS-induced microglia-derived release of proinflammatory factors such as TNF-*α*, IL-1*β*, IL-6, and NO and attenuated LPS-induced nicotinamide adenine dinucleotide phosphate oxidase activation and subsequent ROS production [30, 31]. THSG failed to suppress I*κ*B-*α* degradation, NF-*κ*B phosphorylation and nuclear translocation, and ERK1/2, JNK, and p38 phosphorylation. However, THSG markedly reduced the binding of NF-*κ*B to its DNA element in the iNOS promoter [31]. Moreover, THSG stimulates the secretion of the glial cell-line derived neurotrophic factor and the secretion of brain-derived neurotrophic factor and NGF in cultured rat primary astroglial cells, by activating the ERK1/2 pathway [32].

### 4.3. Alzheimer and Parkinson Diseases

In chronic aluminum exposure or amyloid-*β*(_1–42_)-injected rat models, THSG improves cognitive impairment evaluated using passive avoidance task or Morris water maze tests. THSG reverses the rise in amyloid precursor protein (APP) expression and the downregulation in Src and NR2B mRNA and protein levels in the rat hippocampus [33, 34]. In APP transgenic mouse models, THSG also reverses the increase in *α*-synuclein expression and aggregation in the hippocampus at the late stage of transgenic mice [35].

In 1-methyl-4-phenyl-1,2,3,6-tetrahydropyridine-treated C57BL/6 mouse models of Parkinson disease, THSG protects dopaminergic neurons from degradation in substantia nigra tyrosine hydroxylase-positive cells, enhances striatal dopaminergic transporter protein levels, and increases striatal Akt and GSK3*β* phosphorylation and the upregulation of the Bcl-2/BAD ratio. Furthermore, in the pole test, THSG reduces the times required to turn the body and climbing down to the floor [36].* In vitro*, THSG protects PC12 cells and SH-SY5Y cells against MPP+-induced neurotoxicity. The antiapoptotic effects of THSG were probably mediated through the inhibition of ROS generation and modulation of JNK activation [37, 38], involving activation of PI3K-Akt pathway [39].

### 4.4. Cerebral Ischemia

Previous studies have shown that THSG significantly decreases the percentage of apoptotic cells in injured rat brain tissue induced by ischemia reperfusion, promotes Bcl-2, and inhibits Bax protein expression in brain tissue [40]. THSG also promotes changes in animal nerve behavior; improves neurological function scores; increases the expression of NGF, growth-associated protein 43, and PKA catalytic subunit proteins; and presents a positive correlation between neurological function scores and determined protein expression [41]. In the middle cerebral artery occlusion (MCAO) models, THSG significantly reduces the brain infarct volume and the number of apoptosis cells in the cerebral cortex according to a TUNEL assay [42]. Furthermore, the authors used an* in vitro* ischemic model of oxygen-glucose deprivation followed by reperfusion (OGD-R), revealing that THSG reverses intracellular ROS generation and mitochondrial membrane potential dissipation and inhibits c-Jun N-terminal kinase (JNK) and Bcl-2 family-related apoptotic signaling pathway. Concurrently, THSG prevents the expression of iNOS induced by OGD-R through the activation of SIRT1 and inhibition of NF-*κ*B [42].

## 5. Diabetes and Other Diseases

### 5.1. Diabetes

The beneficial effects of THSG in alleviating diabetic complications are reflected in diabetic nephropathy and gastrointestinal disorders. Treatment with THSG reduces the increase in total cholesterol and triglyceride levels of diabetic rats [43]. Treatment with THSG also significantly reduces blood urea nitrogen, creatinine, 24 hours urinary protein levels, the ratio of kidney weight/body weight, and MDA and markedly increases the activities of SOD and GSH-Px in diabetic rats. Furthermore, THSG inhibits diabetes-induced expression of TGF-*β*1 and cyclooxygenase-2 and restores the reduction of SIRT1 expression in diabetic nephropathy [43]. For disorders of gastrointestinal function in diabetes, long-term preventive treatment with THSG relieves delayed gastric emptying and increases intestinal transit, impaired nonadrenergic-noncholinergic relaxations, and deficiency of neuronal NO synthase expression in streptozotocin-induced diabetic mice. Moreover, THSG prevented significant decreases in PPAR-*γ* and SIRT1 expression in diabetic ileum [44].

### 5.2. Bone Mineral Density

Recently, a study reported that THSG promotes bone mineral density and bone strength in the femoral bones of rats and enhances the bone mineral weight and bone mineral size in the iliac and humeral section after 90 days of administration [45]. Another report described in greater detail how* in vitro* THSG significantly enhances the cell survival, alkaline phosphatase (ALP) activity, and calcium deposition in H_2_O_2_-injured osteoblastic MC3T3-E1 cells. THSG enhances mRNA expressions of ALP, collagen I, and osteocalcin but weakens the receptor activator of nuclear factor-*κ*B ligand and IL-6, as well as intracellular ROS and MDA production [46].

### 5.3. Hair Growth

A report indicated that a THSG fed group had significantly more hair growth compared with the control group, and that THSG accelerated the growth rate of early hair in C57BL/6J mice.* In vitro*, THSG also promoted hair growth in the cultured tentacles follicles of mice, with longer hair than that in the control group after 8 days [47]. Another report indicated that* in vitro* THSG increased the proliferation of dermal papilla cells of mice compared with the control group [48]. In addition, THSG promoted tyrosinase activity and melanin biosynthesis dose-dependently [49, 50].

## 6. Summary

**Figure 2:**
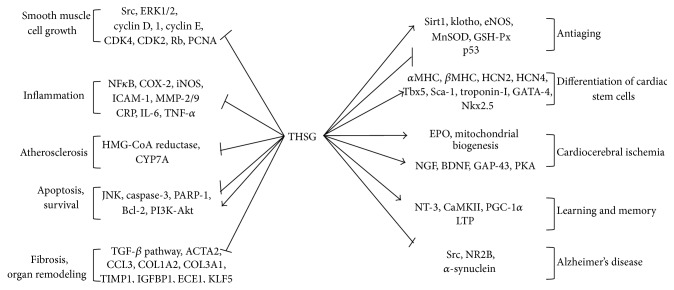
The signal transduction pathways regulated by THSG in the antiaging and aging-related diseases. THSG displays different activities in blocking and activating signaling and gene expression* in vitro* and* in vivo*.

Although THSG has been found to exhibit many medicinal properties, because no systematic study has investigated its regulatory mechanisms and proteomics or genomics data, its functional targets remain unclear. Nevertheless, we summed up the signal transduction pathways that are regulated by THSG, shown in Figure 2, which presents multipathway multitarget characteristics that block and activate different signaling and gene expression. In all the animal experiments in this study, the rats and mice were the main models (Table 1). However, the experiments involving the genetic model and the specific gene knockout model were used less. Most experimental drug dosages of THSG are between 20 and 120 mg/kg, with some individual extreme doses of 300 mg/kg or more. In most studies, THSG has been administered daily by oral gavage, but in some cases it has been delivered by intraperitoneal injection. The pharmacologic activity of THSH in low concentration in cellular studies is summarized in this review (Table 2). Dosages of THSG* in vitro* are normally between 0.1 and 100 *μ*mol/L, whilst in some dosages the concentration will reach a maximum of 300 *μ*mol/L. Then the high concentration of THSG may play a role in toxicological effects instead of activation effects. Because of this, clinical value may be restricted.

From the perspective of drug effects, THSG achieves favorable results in delaying senescence and in treating aging-related diseases, especially in the cardiovascular and nervous system. Some studies have shown that THSG may be more effective than resveratrol in delaying senescence. Nevertheless, more research is necessary to explain the mechanism of THSG.

## Figures and Tables

**Table 1:** Summary of animal experiments of THSG.

Classification	Diseases	Animals	Sex	Induction	Treatment	Duration	Dosage	Administration	Evaluation	Reference number
Antiaging	Vascular senescence	SHRs rats	Male	Genotype	Posttreatment	14 weeks	50 mg/kg	Oral gavage daily	SA-*β*-gal stain; blood flow assay; p53 and phospho- *γ*H2AX determination	[4]
	Senescence	SAMP8 mice	Male	Genotype	Posttreatment	30 days 70 days	2, 20, or 50 *μ*M	Water ad libitum	SA-*β*-gal stain; Morris water maze assay; lifespan assays	[5]
	Senescence	Kunming mice	Male	D-galactose	Posttreatment	4 weeks 8 weeks	42, 84, or 168 mg/kg	Oral gavage daily	Morris water maze assay; Klotho expression in cerebrum, heart, kidney, testis, and epididymis tissues	[6]
	Longevity	*C. elegans*	Male/female	Genotype	Posttreatment	10 hours	50 or 100 *μ*M	Culture liquid	Lifespan assays	[7]
	Dermal thinning	Kunming mice	Male	Natural aging	Posttreatment	8 weeks	18 mg/kg	Oral gavage daily	Dermal layer thickness determination	[51]

Atherosclerosis	Atherosclerosis	NZW rabbits	Male	High cholesterol diet	Posttreatment	12 weeks	25, 50, or 100 mg/kg	Oral gavage daily	Atherosclerotic plaque area; plasma cholesterol; LDL cholesterol; VLDL cholesterol; plasma triglyceride.	[8]
	Vascular dysfunction	SD rats	Male	Atherogenic-Diet	Posttreatment	12 weeks	30, 60, or 120 mg/kg	Oral gavage daily	Vascular reactivity study; eNOS, CRP, IL-6, and TNF-*α* expression	[9, 10]

Myocardial ischaemia	Cardiac ischemia-reperfusion	Wistar rats	Male	Occluding left anterior descending coronary artery	Pretreatment	10 min before reperfusion	7.5 mg/kg	Intravenous injection	ST segment recovery; myocardial infarct size	[16]
	Cardiac ischemia-reperfusion	C57BL/6J mice	Male	Doxorubicin-induced cardiomyopathy	Posttreatment	1 week	10, 30, or 90 mg/kg	Ad libitum	Myocardial mitochondrial biogenesis, improving cardiac function; EPO expression	[17]

Cardiovascular organ remodeling	Vascular injury	SD rats	Male	Carotid arterial balloon injury	Posttreatment	2 weeks	30, 60, or 120 mg/kg	Oral gavage daily	Carotid neointimal formation; PCNA, a-SMA, PDGF-BB gene expression; VSMCs proliferation and migration.	[14]
	Vascular remodeling and fibrosis	SHR rats	Male	Genotype	Posttreatment	14 weeks	50 mg/kg	Oral gavage daily	Intima-media thickness in the aortas, remodeling- related mRNA expressions, and effect on Smad3 deacetylating	[15]
	Cardiac remodeling	SD rats	Male	Pressure-overloaded rats induced by abdominal aortic banding	Posttreatment	30 days	30, 60, or 120 mg/kg	Oral gavage daily	Heart weight and left ventricular weight indexes, MMPs, TIMPs, collagens, TGF-*β*1 protein, ERK1/2, JNK, and p38 activation	[18]

Lipid metabolism	Serum cholesterol	SHR rats	Male	Genotype	Posttreatment	4 weeks	0.15% THSG in rodent chow	Ad libitum	Cholesterol and neutral lipid content VLDL and HDL fraction	[20]
	Serum cholesterol	SD rats	Male	20% lard, 10% cholesterol, and 0.2% propylthiouracil	Posttreatment	1 week	90, 180 mg/kg	Oral gavage daily	Serum TC, TG, LDL- and HDL-cholesterol levels, and LDL receptor mRNA expression	[21]

Learning and memory	*β*-amyloid peptide- or D-galactose- induced dementia	BALb/c mice	Female	Intracranial injection of 3 *μ*L *β*-amyloid_1–40_ or subcutaneous injection of 50 mg/kg D-galactose for 60 days	Posttreatment	60 days	33, 100, or 300 mg/kg	Oral gavage daily	Morris water maze assay; passive avoidance test; MAO-B activity in the cerebral cortex; NGF and NT-3 expression in hippocampal CA1 region	[25, 26]
	Ischemia- reperfusion	Gerbils	Male	Ischemia-reperfusion	Posttreatment	7 days	1.5, 3, or 6 mg/kg	Intraperitoneal injection	Morris water maze test	[27]
	Stress; aging; brain damage	C57BL/6J mice	Male	Sleep-deprived; amyloid-*β*-injected; kainic acid-injected brain damage	Posttreatment	3 days; 17 days and 24 days; 2 weeks	50, 100, or 200 mg/kg	Ad libitum	Passive avoidance task; erythropoietin, PGC-1*α*, and haemoglobin expression	[29]

Alzheimer's and Parkinson's diseases	Alzheimer's disease	SD rats	Male	Chronic aluminum exposure	Posttreatment	1, 3, or 5 months	4 g/kg	Oral gavage daily	Passive avoidance task or Morris water maze tests; APP	[33]
	Alzheimer's disease	SD rats	Male	Amyloid-*β*(_1–42_)-injected	Posttreatment	4 weeks	25 mg/kg	Oral gavage daily	Passive avoidance task or Morris water maze tests; synaptic structures; Src and NR2B expression	[34]
	Alzheimer's disease	APP Tg mice	Male	APPV717I Tg mice	Posttreatment	6 months	120 or 240 *μ*mol/kg/d	Oral gavage daily	*α*-synuclein expression and aggregation in the hippocampus	[35]
	Parkinson's disease	C57BL/6 mice	Male	MPP^+^-induced damage	Posttreatment	14 days	20 or 40 mg/kg	Oral gavage daily	Pole test; tyrosine hydroxylase-positive neurons in the substantia nigral compacts	[36]

Cerebral ischemia	Cerebral ischemia	SD rats	Male	Middle cerebral artery occlusion	Posttreatment	7 days prior to surgery	30, 60, or 120 mg/kg	Oral gavage daily	Percentage of apoptotic cells in injured rat brain tissue; Bcl-2 and Bax protein expression in brain tissue	[40]
	Cerebral ischemia	SD rats	Male	Middle cerebral artery occlusion	Posttreatment	7 days prior to surgery	60 or 120 mg/kg	Oral gavage daily	Animal's nerve behavior and neurological function score; expression of NGF, GAP-43, and PKA catalytic subunit proteins.	[41]
	Cerebral ischemia	Mice	Male	Middle cerebral artery occlusion	Posttreatment	At the onset of reperfusion	15 or 40 mg/kg	Intraperitoneal administration	The brain infarct volume and the number of positive cells	[42]

Diabetes	Diabetic nephropathy	SD rats	Male	60 mg/kg streptozotocin intraperitoneal injection	Posttreatment	8 weeks	10 or 20 mg/kg	Treatment with TSG	Blood urea nitrogen, creatinine, 24 h urinary protein, ratio of kidney weight/body weight, SOD and GSH-Px activities, and TGF-*β*1 and COX-2 expression.	[43]
	Diabetic gastrointestinal dysmotility	Kunming mice	Male	150 mg/kg streptozotocin intraperitoneal injection	Posttreatment	8 weeks	10, 30, or 60 mg/kg	Oral gavage daily	Gastric emptying, intestinal transit, and NANC relaxations	[44]

Bone	Bone mineral density and bone strength	SD rats	Male and female	Natural development (110 ± 10 g)	Posttreatment	90 days	150, 300, or 600 mg/kg	Oral gavage daily	Bone mineral density and bone strength; bone mineral weight and bone mineral size	[45]

Hair	Hair growth	C57BL/6J mice	Female	Natural development (20–26 g)	Posttreatment	9, 18 days	50, 100, or 150 mg/kg	Oral gavage daily	Hair follicles and capillary growth	[47]

**Table 2:** Summary of experiments of THSG *in vitro*.

Classification	Model	Cell types	Induction	THSG concentration	Potential targets or/and pathway	Reference number
Antioxidation	ROS accumulation	3T3 cells; MCF-7	Doxorubicin on MCF-7	60, 120, 180, and 240 *μ*mol/L	SOD; ROS; MitoSOX	[52]
	Apoptosis; ROS accumulation	Human umbilical vein endothelial cells (HUVECs)	Lysophosphatidylcholine (LPC)	0.1, 1, and 10 *μ*mol/L	Caspase-3, Bcl-2, PARP-1, Bax, cytochrome C, SOD, glutathione peroxidase, and MDA	[14]

Cardiovascular protection	VSMCs migration	Vascular smooth muscle cells (VSMCs)	Tumor necrosis factor *α* (TNF-*α*)	0.1–100 *μ*mol/L	Vimentin, TGF*β*1, TGF*β*R1, and Smad2/3	[53]
	Endothelial dysfunction	HUVECs	TNF-*α*	1, 10, 25, 50, and 100 *μ*mol/L	Vimentin, TGF*β*/Smad signaling, TGF*β*1, phosphorylation of Smad2 and Smad3, and nuclear translocation of Smad4	[54]
	Cardioprotection	Primary rat cardiomyocytes	Doxorubicin	10–300 *μ*mol/L	Apoptosis pathway; ROS generation; mitochondrial membrane potential loss; intracellular [Ca^2+^]	[55]
	Endothelial dysfunction	HUVECs	Oxidized low-density lipoprotein (oxLDL)	1, 10, 25, 50, and 100 *μ*mol/L	Vimentin, ICAM-1, VCAM-1, TGF*β*1, phosphorylation of Smad2 and Smad3, and nuclear translocation of Smad4, TGF*β*/Smad pathway; caspase-3 activation	[56]
	VSMCs proliferation	VSMCs	Angiotensin II (Ang II)	1, 10, 25, 50, and 100 *μ*mol/L	Phosphorylated ERK1/2, MEK1/2, and Src; c-fos, c-jun, and c-myc; intracellular ROS; Src-MEK1/2-ERK1/2 signal pathway	[16]
	Cardiac fibroblast proliferation	Primary rat cardiac fibroblast	Ang II; hydrogen peroxide	3–100 *μ*mol/L; 30 *μ*mol/L	ROS-extracellular signal-regulated kinase 1/2 pathway; ERK1/2 activation; MMP-2; MMP-9; MEK	[57]
	Endothelial dysfunction	937 cells	Ox-LDL	30, 60, and 120 *μ*g/L	ICAM-1; VCAM-1	[58]
	VSMCs proliferation	VSMCs	Platelet-derived growth factor- (PDGF-) BB	0.1, 1, 10, and 100 *μ*mol/L	NO-cGMP/PKG pathway	[59]
	VSMCs proliferation	VSMCs	PDGF-BB	1–50 *μ*mol/L	ERK1/2	[15]
	VSMCs proliferation; oxidation of lipoprotein	Porcine coronary arterial smooth cells (CASMCs)	LDL, VLDL, ox-LDL, and ox-VLDL	0.1–100 *μ*mol/L	Oxidation of lipoprotein, proliferation, and decrease of NO content	[60]
	Inflammation	RAW 264.7 macrophage cells	Lipopolysaccharide (LPS)	1, 10, and 100 *μ*mol/L	COX-2	[61]
	Endothelial dysfunction	ECV304	LPC	10 *μ*mol/L	Vascular endothelial growth factor (VEGF)	[62]
	Cardiac stem cells (CSCs) proliferation	Rat CSCs	—	1, 10, and 100 *μ*mol/L	VEGF; T-box transcription factor (Tbx5), hyperpolarization-activated cyclic nucleotide-gated 2 (HCN2), hyperpolarization-activated cyclic nucleotide gated 4 (HCN4), alpha myosin heavy chain (*α*MHC), beta myosin heavy chain (*β*MHC), stem cell antigen 1 (Sca-1), cardiac troponin-I, GATA-4, Nkx2.5, and connexin 43 protein	[22]
	Normal cells	Primary hepatocytes; primary cardiomyocytes; C2C12 myoblasts	—	1.5, 6, 25, and 100 *μ*mol/L	EPO-EPOR; mitochondrial activity and Hb production	[20]

Lipid metabolism	Steatosis hepatic cell	Steatosis hepatic L02 cell	—	50, 100, and 300 *μ*mol/L	HMG-CoA reductase; DGAT1; CYP7A; lipolysis	[12]

Learning and memory	—	Astrocytes; PC12 cells	—	0.4, 2, and 10 *μ*g/mL	Erythropoietin; PPAR-*γ* coactivator 1*α* (PGC-1*α*); haemoglobin-*β*	[29]
	Neurotoxicity	Rat hippocampal neurons	Staurosporine	200 *μ*mol/L	PI3K/Akt signaling; mitochondrial apoptotic pathways	[63]
	Neuroinflammation	Mouse microglial BV2 cell lines	LPS	20–80 *μ*mol/L	NF-*κ*B signaling pathway; ROS production and NADPH oxidase activation	[30]
	Neuroinflammation	Mouse microglial BV2 cell lines	LPS	1, 10, 30, 50, and 100 *μ*mol/L	iNOS; reducing the binding activity of NF-*κ*B	[31]
	Cell model of Parkinson's disease	Human dopaminergic neuroblastoma SH-SY5Y cells.	1-Methyl-4-phenylpyridinium (MPP+)	3.125, 6.25, 12.5, 25, and 50 *μ*mol/L	ROS; mitochondrial membrane potential; the ratio of Bax to Bcl-2; caspase-3; apoptosis	[38]
	Differentiation of PC12 cells	PC12 cells	—	1, 5 *μ*mol/L	MEK and ERK signaling pathways; calcium, CaMKII	[28]

Parkinson's disease	—	PC12 cells	MPP+	0.1, 1, and 10 *μ*mol/L	PI3K/Akt signaling pathway; apoptotic	[39]
	—	PC12 cells	MPP+	1, 5, and 10 *μ*mol/L	ROS generation; JNK	[37]

Bone	Oxidative stress	Osteoblastic MC3T3-E1 cells	Hydrogen peroxide	0.1, 1, and 10 *μ*mol/L	ALP; OCN; COL-I; RNAKL; IL-6; MDA; calcium	[46]

Platelet	Platelet aggregation, secretion	Platelets	Collagen; thrombin; U46619; ADP	10, and 50 *μ*mol/L	Platelet Fc *γ* RIIa, Akt (Ser473), and GSK3*β*(Ser9) phosphorylation.	[24]

Pigmentation	Induction of pigmentation	B16F1 melanoma cells	—	10 *μ*g/L	Microphthalmia-associated transcription factor (MITF); cAMP response element (CRE) binding protein (CREB) activation; p38 MAPK pathway	[50]
	Induction of pigmentation	B16 melanoma cells	—	0.1–12.5 *μ*g/mL	Murine tyrosinase	[49]

## Abbreviations

ADP: Adenosine diphosphate
ALP: Alkaline phosphatase
Ang II: Angiotensin II
APP: Amyloid precursor protein
BDNF: Brain-derived neurotrophic factor
CaMKII: Calcium/calmodulin-dependent protein kinase II
CASMC: Coronary arterial smooth cell
CDK: Cyclin-dependent kinases
COX-2: Cyclooxygenase-2
CRP: C-reactive protein
CYP7A: Cholesterol 7 alpha-hydroxylase
CSC: Cardiac stem cells
DAF-16: A homologous protein of Forkhead box protein O in C. elegans
DAT: Dopaminergic transporter
eNOS: Endothelial NO synthase
EPO: Erythropoietin
ERK1/2: Extracellular signal-regulated kinase 1/2
GAP-43: Growth associated protein 43
GDNF: Glial cell-line derived neurotrophic factor
GPIIb-IIIa/PAC-1: Glycoprotein IIb/IIIa
GSH-Px: Glutathione peroxidase
HCN2: Hyperpolarization-activated cyclic nucleotide-gated 2
HDL: High-density lipoprotein
*γ*H2AX: Histone H2AX phosphorylated on serine 139
HMG-CoA: 3-Hydroxy-3-methylglutaryl-coenzyme A
HUVECs: Human umbilical vein endothelial cells
ICAM-1: Intercellular adhesion molecule-1
IGF-1: Insulin-like growth factor-1
iNOS: Inducible NO synthase
JNK: c-Jun N-terminal kinase
LDL: Low-density lipoprotein
LTP: Long-term potentiation
LPC: Lysophosphatidylcholine
LPS: Lipopolysaccharide
MAO-B: Monoamine oxidase B
MCAO: Cerebral artery occlusion
MDA: Malondialdehyde
MMP: Matrix metalloproteinase
MPO: Myeloperoxidase
MPP+: 1-Methyl-4-phenylpyridinium ion
MPTP: Ethyl-4-phenyl-1,2,3,6-tetrahydropyridine
NADPH: Nicotinamide adenine dinucleotide phosphate
NANC relaxation: Nonadrenergic-noncholinergic relaxation
NF-kappaB: Nuclear factor *κ*B
NGF: Nerve growth factor
nNOS: Neuronal NO synthase
NO: Nitric oxide
NO_*x*_: Nitric oxide and nitrogen dioxide (NO and NO_2_)
NT-3: Neurotrophic factor-3
OGD-R: Oxygen-glucose deprivation followed by reperfusion
PAI-1: Plasminogen activator inhibitor-1
PARP-1: Poly(ADP-ribose) polymerase 1
PCNA: Proliferating cell nuclear antigen
PDGF-BB: Platelet-derived growth factor-BB
PGC-1*α*: PPAR-*γ* coactivator 1*α*
PLC: Lysophosphatidylcholine
PPAR-*γ*: Peroxisome proliferator activated receptor gamma
RANKL: Receptor activator of nuclear factor-*κ*B ligand
ROS: Reactive oxygen species
SA-*β*-gal: Senescence-associated *β*-galactosidase
SAMP8: Senescence-accelerated prone mouse
*α*-SMA: *α*-smooth muscle actin
SOD: Superoxide dismutase
Tbx5: T-box transcription factor
THSG: 2,3,5,4′-Tetrahydroxystilbene-2-O-*β*-D-glucoside
TGF-*β*1: Transforming growth factor beta 1
TNF-*α*: Tumor necrosis factor *α*
TUNEL assay: Terminal deoxynucleotidyl transferase mediated dUTP nick end labeling assay
VCAM-1: Vascular cell adhesion molecule 1
VEGF: Vascular endothelial growth factor
VLDL: Very-low-density lipoprotein
VSMCs: Vascular smooth muscle cells.
